# Linking gender, extramarital affairs, and HIV: a mixed methods study on contextual determinants of extramarital affairs in rural Tanzania

**DOI:** 10.1186/s12981-018-0199-6

**Published:** 2018-06-07

**Authors:** Sally M. Mtenga, Constanze Pfeiffer, Marcel Tanner, Eveline Geubbels, Sonja Merten

**Affiliations:** 1 Ifakara Health Institute (IHI), Ifakara, Tanzania; 2 Swiss Tropical and Public Health Institute (Swiss TPH), Basel, Switzerland; 3 University of Basel, Basel, Switzerland

**Keywords:** Gender, HIV, Context, Microfinance, Masculinity, Extramarital affairs, Tanzania

## Abstract

**Background:**

Extramarital sex is a potential driver of human immunodeficiency virus (HIV) transmission for long-term couples in sub-Saharan Africa. It is increasingly recognized that preventing sexual risk behaviours requires an understanding and adjustment of sexual relationship factors beyond the individual level. We investigated the association between extramarital affairs and HIV status, factors associated with extramarital affairs, and created insights in the context and pathways for married men and women in rural Tanzania who engage in extramarital affairs.

**Methods:**

A cross-sectional sequential explanatory mixed method design was employed. The WHO-Social determinants of health perspective guided the study. Using logistic regression, we analysed the MZIMA project community surveillance representative sample of 3884 married partners aged 15+ residing in Ifakara town, Tanzania (2012–2013). Multinomial logistic regression analysis established the relative risk ratio (RRR) of different social and economic factors with lifetime (proxy) and recent (12 months prior to survey) extramarital affairs. Logistic regression analysis determined the association between extramarital affairs and HIV status. Semi-structured interviews and focus group discussions explored the quantitative findings, capturing the experiences and norms regarding extramarital affairs.

**Results:**

We found a significant association between lifetime (proxy) extramarital affairs and HIV infection among women only. The RRR of having extramarital affairs (lifetime proxy) was significantly higher among Village Community Bank (VICOBA) members, the re-married, consumers of alcohol, those from southern regions, non-Muslims, and those with older age. In the case of recent extramarital affairs (12 months prior to survey), associations were significant for the same variables except for religion, having an income was also associated with the outcome. Qualitative narratives reflected that, desire to prove manhood (masculinity) supported by societal normative beliefs such as; ‘it is not realistic for a man to stay without extramarital partner’ and religious beliefs; ‘a man shall dominate a woman’ encouraged men’s extramarital affairs. For women, striving for financial autonomy, obligations to pay back debts borrowed from several VICOBA, and limited support from their husbands encouraged their engagement in extramarital affairs. Low relationship quality (conflict and sexual dissatisfaction) were reported to encourage both men and women’s extramarital affairs.

**Conclusions:**

The findings show that the link between extramarital affairs and HIV has a gender dimension in which women are more likely to acquire HIV through extramarital affairs (case of recent extramarital affairs (12 months prior to survey). Future programs seeking to address risk sexual behaviors in Tanzanian marriages can consider context-sensitive interventions which address aspects beyond ‘individual risk’ and women’s financial uncertainties, and include couple’s relationship quality, excessive alcohol behaviors, normative masculinity ideology and societal norms, that encourage women’s economic dependence and men’s engagement in multiple sexual partnerships. Microfinance projects (e.g. VICOBA) could be a platform for gender-transformative approaches, combining economic empowerment and HIV risk protection strategies.

## Background

Extramarital sex is a potential driver of increased risk of human immune virus (HIV) transmission among couples in long-term relations in sub-Saharan Africa (SSA). An incidence study among stable couples in SSA estimates that 22.5% (range 11.1–39.8%) of HIV infections are acquired by one of the partners from sources external to the couple [1], with multiple partnerships having contributed to HIV transmission both during early and advanced stages of the HIV epidemic [2]. Absence of contextually adapted information to address extramarital affairs in marriage, together with an underestimation of the potential risk of this behavior among married partners [3] are likely to have undermined the effectiveness of proven HIV interventions in the region.

In Tanzania, as elsewhere in sub-Saharan Africa (SSA) various studies suggest that extramarital sex is common among married and stable couples [4]. About 70% of participants from a study in urban and rural districts in Tanzania reported ever having engaged in an extramarital affair, and that extramarital affairs were identified as one of the main driver of HIV transmission [5].

Whether engaging in concomitant sexual relationships involves married or unmarried individuals is making a difference with regards to HIV prevention [6]. This is because, unlike unmarried partners, married partners may feel protected from sexually transmitted infections, as it is not expected to have other sexual partners outside the union. Hence, it is likely that married partners may underestimate their spouse’s potential of engaging in risky sexual behaviors including having multiple sexual partners. This is especially problematic as being in a marriage usually constrains partners’ adoption and communication about HIV prevention interventions including condom use and couple counseling and testing, due to aspects related to marital norms, gender power relation and relationship quality [7, 8]. In some communities in Tanzania, women may not be allowed to suggest safer sex use to their husbands in marriage as this may be considered as against societal expectations about married partners [8]. A study in Zambia also found that married women are often not given the right to decide on HIV health matters [9]. This is likely to heighten the risk for HIV transmission since partners perceive themselves at a low risk of HIV infection [10], do not realize that a partner may be infected with HIV [11], and are less likely to use protection during extramarital affairs [6]. Therefore, being in a marriage with someone who has other partners outside the union subjects both partners to the risk of HIV infection.

Various social-behavioral aspects have been associated with extramarital affairs in SSA. In Tanzania, a study among men shows that, higher education, being older, a lower age at first sexual intercourse, and having sex before marriage were significantly associated with extramarital affairs [12]. Agnarson in Tanzania also found that concurrent partnerships are sometimes a way to assure financial security for women [13]. In Nigeria the number of sexual partners before marriage, polygamy, wealth, being in a monogamous marriage, the region, and self-perceived risk of HIV infection were linked to extramarital affairs [14]. In Côte d’lvoire age at debut was significantly associated with extramarital affairs [15].

Individual behavior-change approaches have been consistently employed to address extramarital affairs and HIV vulnerability in marriage. For example, in Tanzania, media campaigns with a Swahili slogan ‘baki njia kuu mchepuko sio dili, epuka ukimwi’ (literally, remain on the main road, divergence is not a deal, avoid HIV) [16] have been widely used to advise married partners to abstain from extramarital affairs. In Tanzania couple counseling and testing services are well implemented and accessible in various levels of the health facilities. In SSA, couple counseling and testing services are known to improve adoption of safer sex practices including condom use and reduction of multiple sexual partners among couples [17, 18].

Increasing recognition that high-risk sexual behaviors and HIV vulnerability are entrenched within the broader social, cultural, economic and political contexts [19–21], requires a wider understanding of how these aspects are likely to shape extramarital affairs in a localized context. However, there is limited contextual information of what contributes to extramarital affairs particularly in Tanzania. This gap is likely to limit appropriate strategies to address the behavior and mitigate risk of HIV transmission among married couples in the country.

Therefore, this study aims at investigating the association between extramarital affairs and HIV status, factors associated with extramarital affairs, as well as understanding the contextual aspects and the pathways by which men and women come to engage in extramarital affairs. We hope that the findings can inform comprehensive HIV prevention approaches for HIV prevention in marriage. We further investigated the association between extramarital affairs and HIV status among married men and women in the respective local context.

### Theoretical framework

**Fig. 1:**
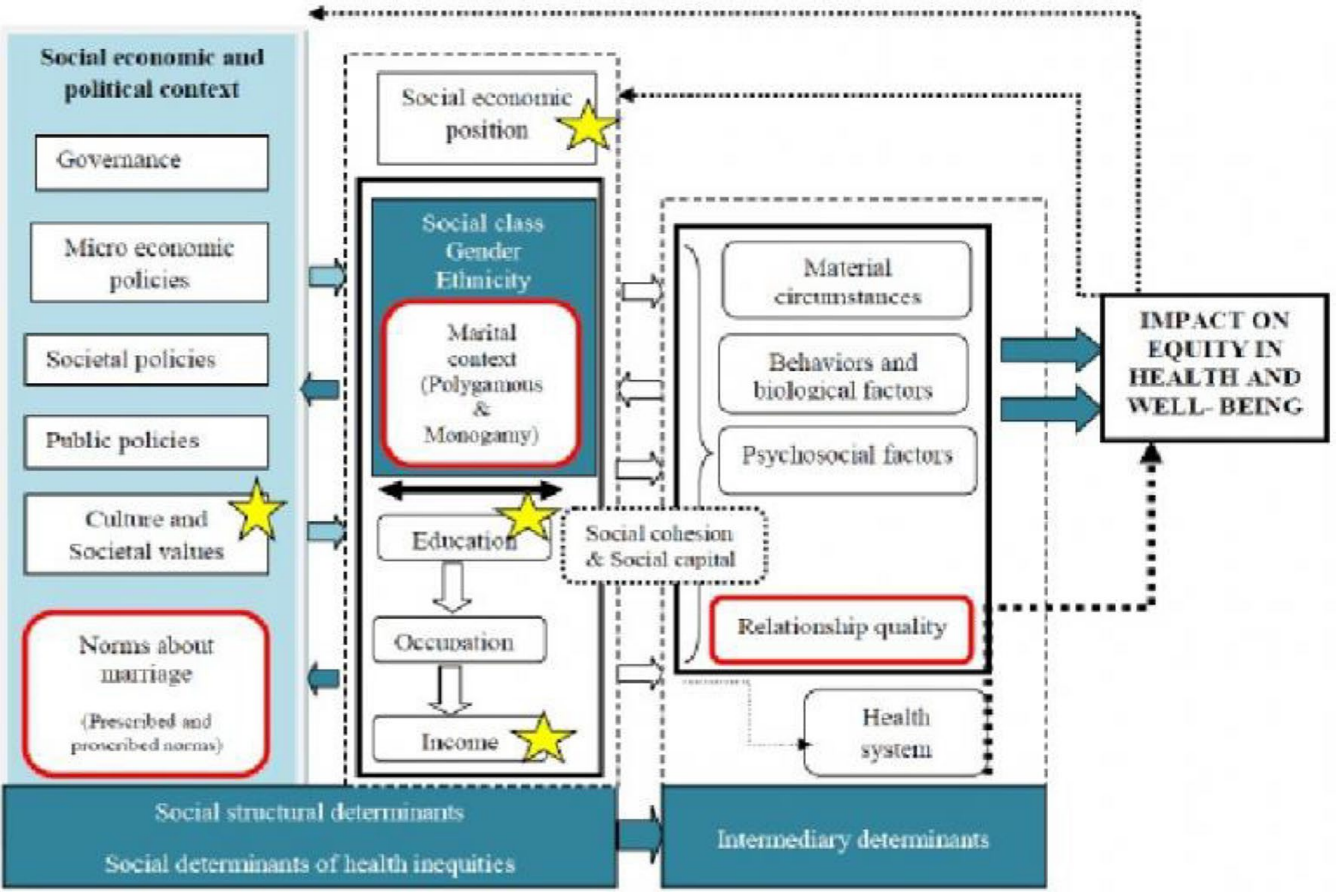
Modified WHO-CSDH framework used to learn about the challenges to safer sex dialogue between married spouses [22]

We used the framework of the World Health Organization’s Commission on the Social Determinants of Health (WHO-CSDH) [22] to contextualize sexual behavior in the wider social-structural environment. The emphasis on contextual determinants distinguishes the WHO-CSDH framework from other psychological and behavioral theories, which focus mostly on attitudes, perceptions, and other individual level determinants. Although, the WHO-CSDH framework is not meant to specifically investigate factors influencing extramarital relations, the framework comprises of broader social-structural aspects that could be adopted to study any health-related condition/behavior. To guide the examination of the social determinants of extramarital affairs as a driver for HIV infection, we used the WHO-CSDH constructs (highlighted in yellow), but we also added constructs (highlighted in red) as illustrated in Fig. 1. The additional constructs (highlighted in red) were based on insights from an earlier study conducted in the same project and context which highlighted their relevance in explaining safer sex communication practices in marriage [8].

## Methods

### Overall study design

A cross-sectional sequential explanatory mixed-method study was employed [23] to understand the contextual determinants of extramarital affairs. The qualitative study followed the quantitative study to explain the quantitative results and further explore men’s and women’s experiences, and normative aspects that relate to extramarital practice. Both studies were carried on in Ifakara town, Kilombero district in southeastern, Tanzania. Ifakara town is heterogeneous, comprising of more than nine ethnic groups coming from various parts of the country [24]. Kiswahili is the main language spoken in the area. Islam and Christianity are the predominant religions. The town comprises a hierarchy of health facilities ranging from the referral hospital to health centers and dispensaries. The adult HIV prevalence in Ifakara based on the MZIMA community surveillance study is 7% (57) which is higher than the national prevalence of 5.2% [24].

### Quantitative methods

A detailed description of the quantitative method for this study is published elsewhere [24]. We provide a brief description here.

### Study setting

The study comprised of married and cohabiting partners who were enrolled in the MZIMA community health closed cohort study implemented between April 2012 and April 2013 in Ifakara town, Kilombero district, Tanzania. MZIMA, which means being healthy in Kiswahili, aimed to establish the prevalence of communicable and non-communicable diseases and their associated social-economic determinants. A detailed description of MZIMA cohort study is published elsewhere [24].

### Study design and sample

This cross-sectional study uses data from the first round of MZIMA cohort study which was captured through a questionnaire that was taken orally in a face-to-face during house visits in which the HIV tests were also performed. All households located within the Ifakara town health and demographic surveillance system were selected. Upon arrival in the selected households, the eligible participants (men and women age 15 years+) were informed, interviewed, and asked to provide biological specimens for HIV testing if they provided written informed consent. During the first round of the MZIMA cohort study 8734 were interviewed, 92.9% of whom provided blood samples for HIV testing. Because we aimed to investigate factors that determine extramarital affairs in stable relationships, in the present study we restricted the sample to 3884 participants who said they were officially married or cohabiting (49.2% of the total study sample). Participants were interviewed in a private place within the premises of the household. The number of persons with extramarital affairs in the past 12 months and of the proxy for extramarital affairs before that was sufficient to perform multivariable multinomial statistical analysis.

### Variables and measures

#### Independent variables

Detail description about how the variables were measured is published elsewhere [24] except for Village Community Bank (VICOBA) membership. Village Community Banks (VICOBA) were initiated in Tanzania as an informal structure to support savings and profit among low income community members. More women than men participate in a VICOBA. VICOBA have evolved and now include people of higher economic status. This variable was included in the study based on the assumption that a VICOBA is an income-securing structure, and members of VICOBA are therefore less likely to be involved in extramarital affairs. VICOBA membership was measured by requesting participants to report on whether they were members of a microfinance program in their community. Participants who reported that they were a member of a VICOBA were assigned a ‘Yes’.

We examined the demographic (sex; age; income; education; ethnicity), social-structural (being part of a Village Community Bank, a ‘VICOBA’; performing any income generating activity), marital characteristics (being re-married or not), health care utilization (VCT access) and lifestyle (alcohol consumption) as correlates of extramarital relationship prevalence. VICOBA membership was measured by requesting participants to report on whether they were members of a microfinance program in their community. Except being part of a VICOBA, these variables were selected based on a review of previous literature [25, 26], the WHO-CSDH framework [27], and on the insights from previous qualitative research.

#### Outcome variables

The study had two outcome variables: HIV status as main, and extramarital affairs as intermediate outcome. First, we establish associations of extramarital affairs with HIV infection stratified by sex of the respondent; second, associations of the independent variables with extramarital affairs were determined. Due to the sensitive nature of the topic, participants were not directly asked whether they engaged in extramarital affairs. The variable ‘extramarital affairs’ was constructed based on marital status and the number of sexual partners (a) in the 12 months prior to the survey, and (b) over the lifetime. As all participants in this study were in a monogamous marriage, participants with more than one sexual partner in the past 12 months were coded as having had extramarital affairs in the 12 months prior to the survey. Because the number of persons who reported extramarital affairs in the last 12 months was low, and with the given HIV incidence less than 1 new HIV infection would be expected in this group, associations of extramarital affairs in the MZIMA sample could not be measured with this variable. Therefore, a variable for extramarital affairs over the course of a person’s marriage was estimated. The overall number of partners of a respondent was available for the respondents, as well as their age at marriage. Based on these variables we estimated the occurrence of lifetime extramarital affairs based on the median age at marriage and the number of partners. At the age of 25 years half of the men were married, whereas among women half of the women were married already at the age of 19. In our sample men who were 25 years old had in average 4.4 partners, while women of age 19 years had 2.8 partners. Since the Tanzanian HIV/AIDS and Malaria Indicator Survey (THMIS) 2011–2012 report shows that men reported more partners than women, this led to the assumption that a woman marries her second partner, whereas a man gets married in average to the fourth partner. Men with less than 5 partners and women with less than 3 partners were considered as not having had extramarital affairs (= 0), whereas it was assumed that if men had 5 or more and women 3 or more partners some of these relationships happened within their marriage (= 1). Therefore, ever having had extramarital affairs is referred here and after as ‘estimated’ or ‘proxy’ lifetime extramarital affairs. For this analysis, extramarital affairs were defined as a trichotomous variable comprising of the values corresponding to the following: 0 = no extramarital affair; 1 = lifetime extramarital affair (proxy based on number of lifetime partners) but not in 12 months prior to survey; 2 = extramarital affairs in 12 months prior to survey.

### Statistical analyses

First, descriptive statistics were conducted to list outcomes for all variables. We conducted bivariate analyses (cross tabulation) to analyze the associations between each of the independent variables and the two categories of ‘extramarital affairs’. The significance of the associations between the dependent and independent categorical variables were tested using the Pearson Chi square test. Bivariate logistic regression analysis was then performed to determine the association between extramarital affairs and HIV status, stratified by sex.

Odds ratios (OR) and 95% confidence intervals (CI) were calculated using logistic regression analysis to assess associations of extramarital affairs and remarriage with HIV status. We then performed multinomial logistic regression to calculate the adjusted relative risk ratio (RRR) and 95% confidence interval (CI) to identify the independent predictors of proxy lifetime and 12 months extramarital affairs. The model used sex, age and level of education as fixed a priori covariates. Selection of variables for the multinomial analyses was based on their ability to improve the overall model using log likelihood ratio test. Statistical interactions of sex and other independent variables were assessed, and consequently the model was stratified for sex of the participant. RRR and their corresponding 95% confidence intervals, and P-values were reported for the final multinomial regression models. Associations were considered statistically significant if the P-value was < 0.05. Predictive margins were calculated for men and women separately. All analyses were performed using STATA version 14 (StataCorp, USA).

### Qualitative methods

Study participants. We targeted sexually active married men and women of age between 18 and 60 years who participated in 24 in-depth interviews (IDIs) and 6 focus group discussions (FGDs).

### Setting and recruitment

The study was implemented between May and August 2015 in two villages where the quantitative participants were recruited in Ifakara town (Viwanja sitini and Mlabani villages). A more description of the recruitment procedure is published elsewhere [8].

### Instrument design

For the IDI we used semi-structured interview guides and for FGD we used an open-ended question guide. Both tools consisted of a series of themes constructed from the quantitative study. However, to gain deeper insights into extramarital practice, the IDIs elicited marital relationship quality and personal experiences regarding extramarital affairs, while the FGDs explored social norms regarding extramarital affairs.

### Data collection

A married female social scientist (SM) with extensive experience in conducting HIV-related qualitative research conducted most of the interviews. One married male research assistant (RA)—a university graduate in sociology with experience in qualitative studies assisted in some interviews and discussion sessions with male participants. Prior to formal interviews, the social scientist conducted the informal discussions with some members of the community to gain insights about the topic and the general marital lifestyle.

The formal interviews and discussions were conducted in Kiswahili, recorded verbatim and subsequently transcribed by the social scientist (SM) assisted with a trained research assistant. We used a vignette (*“There was a couple in village ‘A’. This couple used to be very much in love. They stayed in this way for a very long time. But it reached a time when the man started realizing that his wife was having some affairs with another man. Seeing that his wife was doing that, the husband also began dating other women in the nearby village”*) to make the topic less personal and encourage participants to freely provide their own opinion about extramarital practice. In the vignette, we chose to portray a woman as the first to perform extramarital affairs since unlike men, women may not easily disclose their behaviors regarding engaging in multiple sexual partnership. Privacy and confidentiality of information were ensured.

### Data analysis

The social scientist (SM) independently coded and analyzed the transcribed data thematically [28].

**Fig. 2:**
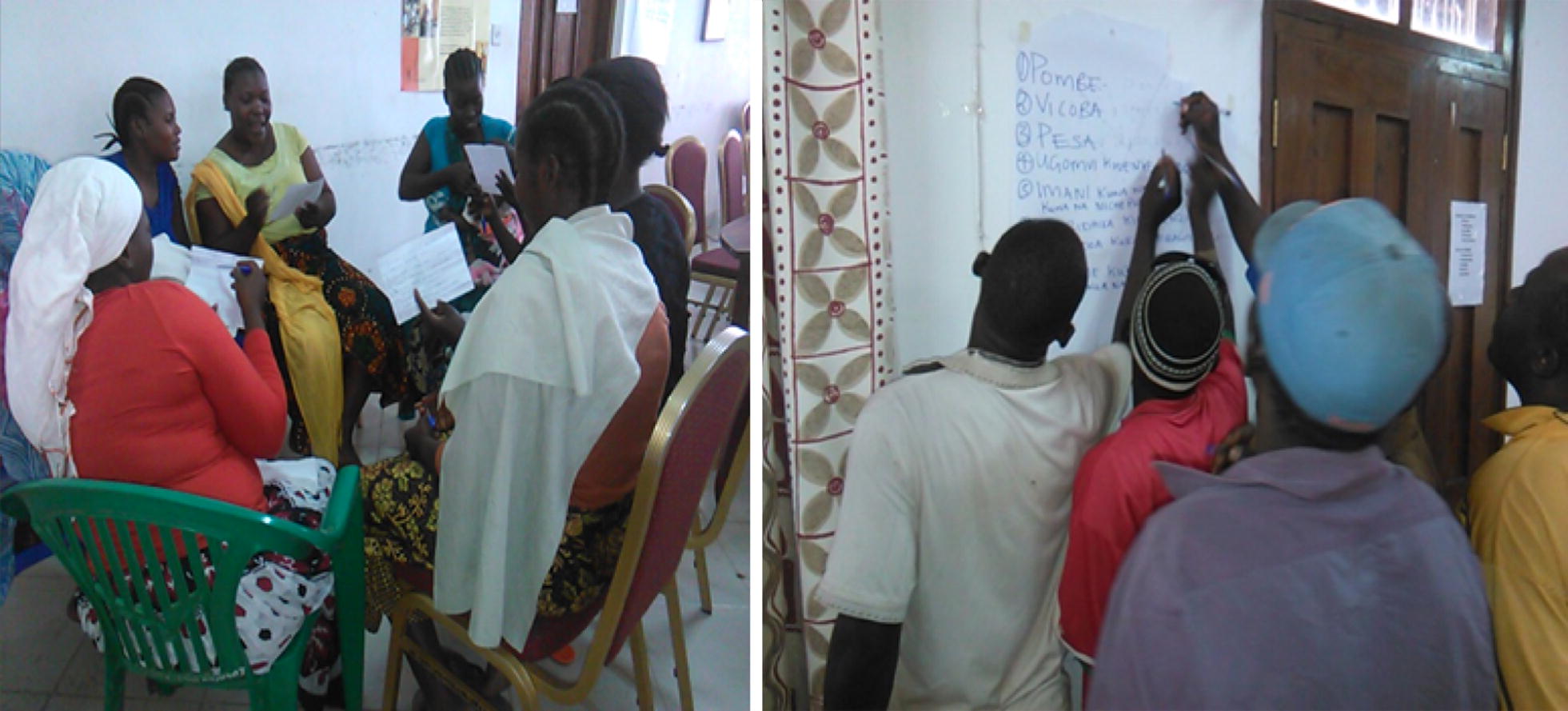
A picture showing participants conducting member check for qualitative data validation

Pre-determined themes drawn from the topic guides (i.e. reasons for practicing extramarital affairs) and from the quantitative findings were generated. Through open coding, emerging themes were subsequently added. To enhance validity, another social scientist assisted in conducting independent coding, the codes were compared, and the discrepancies were resolved. Also, themes developed by the social scientist were cross-checked within the research team. In addition, member checking (Fig. 2) was used to check for accuracy of the coding and interpretation as described in the previous study [8]. N-VIVO program version 12 for qualitative data analysis was used to support rigorous data coding. All data were analyzed in Kiswahili, and relevant quotes were translated into English for this study.

## Results

### Quantitative study

#### Characteristics of study population

Characteristics of survey respondents are summarized in Table 1. There were 3884 married and cohabiting participants of which the majorities (35.2%) were between the ages of 25–34 years, females accounted for 66.4%. Eighty-eight percent of participants had attained the formal education, 92% among males and 84% among females. Sixty-four percent of females and 90% of male respondents performed an income generating activity. More than half of participants (61%) were Christians and 38% were Muslims. Seventeen percent of participants were not living with the person they had married first. About 10% of men and 2.2% women had engaged in extramarital affairs in the 12 months prior to the survey, with percentages for lifetime extramarital affairs (proxy) being estimated to be 45.5 for men and 43.8% for women.

**Table 1:** Profile of respondents for extramarital affairs assessment in Ifakara, Tanzania (n = 3884)

Variable	N males	Percent (%)	N females	Percent (%)	Percent overall (%)
Overall	1305	100	2579	100	3884
Age (years)					
15–24	34	2.6	532	20.6	14.6
25–34	388	29.7	978	37.9	35.2
35–44	344	26.4	534	20.7	22.6
45–54	203	15.6	309	12	13.2
> 54.9	336	25.7	226	8.8	14.5
Education					
Yes (formal education)	1213	93	2199	85.3	87.8
No (formal education)	92	7	380	14.7	12.3
Performing any income generating activity					
Yes	1183	90.7	1649	63.9	72.9
No	122	9.3	930	36.1	27.1
Religion					
Muslim	508	38.9	986	38.2	38.5
Christian-catholic	691	53	1362	52.8	52.9
Other Christian	99	7.6	221	8.6	8.2
Other/none	7	0.5	10	0.4	0.4
Ethnicity					
North west	112	8.6	189	7.3	7.7
North east	50	3.6	75	2.9	3.2
Southern	1143	87.8	2315	89.8	89.0
Ever received VCT?					
No	262	20.1	714	27.7	25.1
Yes	1043	79.9	1865	72.3	74.9
Alcohol consumption					
No	573	43.9	504	19.5	27.8
Yes	732	56.1	2075	80.5	72.3
VICOBA membership					
No	55	4.2	376	14.6	11.1
Yes	1250	95.8	2203	85.4	88.9
Remarried					
Yes	1056	80.9	2172	84.2	83.1
No	249	19.1	407	15.8	16.9
Extramarital relationships (EM)					
Never had EM	590	45.2	1392	54.0	51.0
Proxy-life time EM (excl. past 12 months)	594	45.5	1130	43.8	44.4
EM, past 12 months	121	9.3	57	2.2	4.58
Mean (CI) Nb of partners, never EM	2.5	(2.4–2.6)	1.4	(1.37–1.43)	
Mean (CI) Nb of partners, proxy life-time EM	12.2	(11.2–13.2)	4.7	(4.5–4.9)	
Mean (CI) Nb of partners, EM, past 12 months	14.1	(10.9–17.2)	5.3	(4.2–6.5)	

#### Association between extramarital affairs and HIV infection

Table 2 shows the association between extramarital affairs and HIV infection. Women with estimated lifetime extramarital affairs were more likely to be HIV positive than those who had not had extramarital affairs (OR = 1.55, 95% CI 1.113–2.13). Women who were remarried were also more likely to be HIV positive (OR = 1.50, 95% CI 1.04–2.18). We did not find any statistical significant association between extramarital affairs and HIV among men. We looked at the association between age and HIV but did not find a significant association.

**Table 2:** The Logistic regression results of the association between extramarital affairs and HIV status of men and women in Ifakara town, Tanzania (2015)

	% (n/N)	OR (95% CI)	P-value
Males (n = 1305)			
Extramarital affairs (EM)			
Never had EM (reference cat.)	685	1	–
Proxy-life time EM (excl. past 12 months) (cat. 1)	499	0.86 (0.54–1.37)	0.523
EM, past 12 months (cat. 2)	121	0.38 (0.12–1.28)	0.119
Remarried	249	1.60 (0.95–2.69)	0.074
Female (n = 2579)			
Extramarital affairs (EM)			
Never had EM (reference cat.)	1539	1	–
Proxy-life time EM (excl. past 12 months) (cat. 1)	983	1.55 (1.13–2.13)	0.006
EM, past 12 months (cat. 2)	57	1.33 (0.47–3.76)	0.595
Remarried	407	1.50 (1.04–2.18)	0.031

#### Independent predictors of extramarital affairs

Table 3 presents the results of the multinomial logistic regression analysis. The majority of the independent predictors of the proxy lifetime extramarital affairs were consistent with those in recent (12 months prior to survey) extramarital affairs. The relative risk ratio (RRR) of having extramarital affairs versus non-extramarital affairs in the proxy-lifetime significantly decreased as age increased (RRR: 0.98 95% CI 0.98–0.99), was higher among men (RRR: 1.27; 95% CI 1.06–1.52), among members of a Village Community Bank (VICOBA) (RRR: 1.35; 95% CI 1.08–1.69), among consumers of alcohol (RRR: 1.54; 95% CI 1.29–1.82), among those who re-married (RRR: 2.50; 95% CI 2.07–3.02) and were lower among those of catholic religion (RRR: 0.82; 95% CI 0.71–0.95), and other Christian religion (RRR: 0.52; 95% CI 0.39–0.70) if compared to the Muslim population.

**Table 3:** Multinomial logistic regression of correlates of extramarital affairs in Ifakara, Tanzania (N = 3711)

Covariate	All adults				Males				Females			
	RRR	95% CI		P-value	RRR	95% CI		P-value	RRR	95% CI		P-value
		Lower limit	Upper limit			Lower limit	Upper limit			Lower limit	Upper limit	
Outcome category 1: extramarital affairs in lifetime												
Males (ref = females)	1.27	1.06	1.52	*0.007*	–	–	–	–	–	–	–	–
Age (increment: +10 years)	0.98	0.98	0.99	*0.000*	1.00	0.99	1.01	0.688	0.97	0.96	0.98	*0.000*
Had formal education (ref = no formal education)	1.15	0.90	1.45	0.213	1.00	0.62	1.61	0.997	1.16	0.90	1.51	0.252
Perfom any income activity (ref = doesn’t performs)	1.15	0.97	1.35	0.107	1.47	0.96	2.27	0.073	1.20	0.99	1.45	0.058
Ethnic group (ref = northern western)												
Northern eastern	1.09	0.67	1.77	0.730	1.30	0.62	2.76	0.487	0.83	0.41	1.65	0.590
Southern	1.53	1.14	2.06	*0.005*	0.87	0.54	1.43	0.593	2.08	1.42	3.05	*0.000*
VICOBA membership (ref = non-member)	1.35	1.08	1.69	*0.009*	1.89	0.98	3.70	0.058	1.29	1.02	1.64	*0.037*
Re-married (ref = not re-married)	2.50	2.07	3.02	*0.000*	1.23	0.90	1.68	0.197	3.78	2.96	4.84	*0.000*
Drunk alcohol (ref = never)	1.54	1.29	1.82	*0.000*	1.38	1.08	1.79	*0.011*	1.64	1.32	2.04	*0.000*
Never had VCT (ref = ever)	0.87	0.74	1.03	0.099	0.91	0.67	1.25	0.570	0.86	0.71	1.04	0.131
Religion (ref = Muslim)												
Christian-catholic	0.82	0.71	0.95	*0.008*	0.87	0.67	1.13	0.305	0.80	0.66	0.96	*0.015*
Other	0.52	0.39	0.70	*0.000*	0.69	0.41	1.17	0.172	0.42	0.29	0.61	*0.000*
Other/none	0.42	0.13	1.32	0.138	0.71	0.12	4.38	0.715	0.35	0.07	1.69	0.190
Constant	2.58	1.59	4.19	0.000	2.62	1.02	6.78	0.046	2.46	1.37	4.44	0.003
Outcome category 2: extramarital affairs in past 12 months												
Males (ref = females)	5.26	3.85	7.14	*0.000*	–	–	–	–	–	–	–	–
Age (increment: +10 years)	0.95	0.94	0.96	*0.000*	0.95	0.94	0.97	*0.000*	0.95	0.93	0.97	*0.000*
Had formal education (ref = no formal education)	1.23	0.78	2.0	0.387	1.16	0.86	1.96	0.710	1.54	2.78	0.83	0.172
Performs any income-generating activity (ref = doesn’t performs)	2.70	1.64	4.35	*0.000*	6.25	1.45	25.0	*0.014*	2.17	1.25	3.70	*0.006*
Ethinic group (ref = northern western)												
Northern eastern	1.37	0.62	3.02	0.438	1.31	0.50	3.44	0.579	2.52	0.39	16.30	0.332
Southern	1.23	0.71	2.12	0.462	0.77	0.40	1.48	0.428	3.31	0.78	14.05	0.104
VICOBA membership (ref = non-member)	2.63	1.72	4.0	*0.000*	5.0	2.38	11.11	*0.000*	2.04	1.19	3.45	*0.009*
Remarried (ref = not)	2.41	1.69	3.43	*0.000*	1.27	0.79	2.06	0.328	4.27	2.53	7.21	*0.000*
Drunk alcohol (ref = never)	2.5	1.85	3.33	*0.000*	2.5	1.69	3.70	*0.000*	2.5	1.49	4.1	*0.000*
Ever had VCT (ref = never)	0.87	0.63	1.19	0.373	0.72	0.47	1.09	0.120	1.20	0.72	1.99	0.478
Religion (ref = Muslim)												
Christian-catholic	0.91	0.68	1.23	0.558	0.87	0.59	1.30	0.498	1.05	0.66	1.68	0.830
Other	0.59	0.33	1.08	0.088	0.80	0.38	1.65	0.539	0.25	0.06	1.07	0.061
Other/none	0.99	0.20	4.99	0.988	1.74	0.26	11.60	0.566	0.00	0.00	–	0.978
Constant	9.06	3.67	22.40	0.000	26.56	7.51	93.99	0.000	0.32	0.05	1.94	0.217

Italic values are statistically signiicant (P < 0.05)

Likewise, the relative risk ratio (RRR) of having recent extramarital affairs (12 months prior to survey) versus non-extramarital affairs, significantly decreased with increasing age (RRR: 0.95; 95% CI 0.94–0.96), was higher among those with stable sources of income (RRR: 2.70; 95% CI 1.64–4.35), among the VICOBA members (RRR: 2.63; 95% CI 1.72–4.0), among the re-married (RRR: 2.41; 95% CI 1.69–3.43) and among those consuming alcohol (RRR: 2.5; 95% CI 1.85–3.33). When calculating predictive margins for men and women separately; we found that most associations with extramarital affairs were similar for men and women. Only a few differences can be noted, in the youngest age-category men were less likely to have had extramarital affairs in contrast to women; women from the southern region, were more likely to have extramarital affairs than men; religion played a role mainly for women.

#### Qualitative study

Twenty-four participants (13 females and 11 males) took part in the IDIs (Table 4). Thirty-eight individuals participated in the focus group discussions (18 males, 21 females) (Table 5). Participants were of age between 18 and 68. All participants were currently married, 44 lived in monogamous marriages and 20 in polygamous ones. Most participants reported to have had 1–7 children and had been married for 10 years or less.

**Table 4:** Summary of the IDIs participants in Ifakara town (n = 24)

Participant’s characteristics	Total number of participants
Sex	
Males	11
Females	13
Age	
25–34	09
35–44	08
45–54	06
> 54.9	02
Education	
Never gone to school	01
Primary education	20
Secondary education	02
Higher level	01
Occupation	
Farmers	13
Farmers/business	02
Petty traders	04
Business	04
Teacher	01
Marital type	
Monogamous	15
Polygamous	09
Marital duration	
1–5	09
6–11	06
12–17	05
18–22	03
30	01
Number of children	
0	01
1–3	13
4–6	07
7–9	02
20	01
Religion	
Muslims	07
Christians	17

**Table 5:** Summary of the FGD participants in Ifakara town (n = 38)

Participant’s characteristics	Total number of participants
Sex	
Males	18
Females	20
Age	
18–25	12
26–34	11
35–43	15
Education	
Primary education	36
Secondary education	02
Occupation	
Farmers	25
Petty traders	06
Business	07
Marital type	
Monogamous	25
Polygamous	13
Marital duration	
1–5	25
6–11	09
12–17	04
Number of children	
1–3	27
4–6	11
Religion	
Muslims	15
Christians	23

#### Theme 1: Describing extramarital practice in Ifakara town

‘*Kidumu*’ and ‘*Mchepuko*’ were the local names for extramarital affairs in Ifakara town. In Kiswahili, Kidumu symbolically denote a plastic container, which is used to store local brew, milk and water. Mchepuko is diverging. As such, Kidumu in Ifakara is considered as a backup for sexual desires and economic needs as reflected in the subsequent excerpts.

When the Vignette (refer to the method section) was presented, various participants reported that the extramarital affairs is a common behavior among men and women in the community and that one spouse may initiate the behavior and another spouse may do the same for revenge:

*“Extramarital affairs happen much in our community, we see people, husbands and wives, competing in moving out with other partners, one (spouse) may initiate (extramarital affair) and another one may do the same as revenge, I saw one of my friends doing the same”*

[IDI, Woman_24 years, Monogamous]

#### Theme 2: Participant’s views about the reasons behind extramarital affairs

In this section, emerged themes on the determinants of extramarital affairs are presented based on the social-cultural domains as adopted from the WHO-CSDH framework. From the interpretation point of view, most themes were converging. We thus used broader theoretical domains to categorize the key themes.

### Social-cultural aspects

#### Religion, masculinity and societal norm

Men’s extramarital affairs were linked to religious faith, societal norms and masculinity. From the religious perspective, participant’s views reflected that men’s extramarital affairs is justified based on the notion that, religious books endorse men’s dominance over women: *“Even the religious books stated that a man should dominate his wife and not otherwise and so he ‘can have extramarital affairs’* [IDI_ Male_ 56 years_Polygamous]. Interestingly, while men’s extramarital affairs could be justified based on religious faith, on the other hand, women’s extramarital practice seems not to be justifiable based on cultural system: “*the wife who have extramarital affairs destroys the system of culture”* [IDI_ Male_ 56 years_ Polygamous].

Staying without extramarital affairs is believed to be ‘unrealistic to men’. Some participant’s views reflected a notion of masculinity in a self-reflective attitude, where one participant (man) declared that he cannot stay without having extramarital partner since extramarital affairs for men is believed to be a reality: *“I cannot stay like this without a woman (extramarital partner), it is not realistic for a man, I cannot lie”.* In addition, for men, having fewer or several extramarital partners may depend on the affordability (being good financially):

“*I used to have several (extramarital women) but I now have only one* ***‘kidumu’*** *(extramarital partner)”, […] since I cannot afford (not having money) many at the moment”*

[IDI_Man_26 years_ Monogamous]

Participant’s views also reflected that masculinity norm exemplifies the normalization of men’s extramarital affairs, such that, some ‘repugnant names’ such as ‘prostitute’ associated with having multiple sexual partners are mainly used to refer females and not men:

*“A man […] with several women outside marital will never be called a prostitute, because ‘he is a man’, he can even finish all the women in the village but will never be called a prostitute […] but not you (woman)”*

[IDI_Female_26 year_Monogamous]

#### Village Community Bank (VICOBA) and women’s economic hardship

Women and men frequently cited VICOBA as an aspect that drives women to seek extramarital partners. Participants (mostly women) reported that women’s economic hardship, lack of financial support from their husbands and the obligations to pay back debts to several VICOBA encourage women to initiate and continue with extramarital behavior.

One woman explained her experience:

*F: How long have you been with this “mchepuko” (extramarital partner)?*

*P: 3* *years*

*F: Can you please tell me how the relationship started?*

*P: There is a time that I was supposed to give back the money to VICOBA. But I did not have money, therefore, group members took me to the village chairman. I explained to the village chairman that my husband does not want to assist me in paying the money back to VICOBA (fearing that the woman will be superior in the house), so when I was explaining this to the chairperson there was a man aside who was listening and looking at me. When I was leaving that man followed me and told me that [..] “I will assist you in paying the VICOBA” I said why can’t I have an affair with this man who is ready to help me? Then we started the relationship”*

[IDI_Female_35years_Monogamous]

Clarifications which were provided during ‘member check’ validation session with some community members in the study site highlighted that, some women in Ifakara town prefer to engage in several VICOBA (i.e. a woman could be a member of more than two VICOBA). Those women with poor economic background who are members of more than one VICOBA, are likely to encounter difficulties in financing their membership in all the VICOBA groups. This was mentioned as a common scenario that is likely to expose some women to extramarital affairs.

Benefits attached to extramarital affairs (mainly from women’s views) include receiving support for the various needs such as food and cash for financing the farming activities. It could be that there is a cost–benefit analysis attached to the extramarital practice: *“The extramarital partner assists me a lot, he gives me money for my farming activities, provides food, sometimes he can even buy beans, flour which help me and my child to survive. […] he (husband) does not provide anything for the family [..], all he knows is to drink alcohol [….]”* [IDI_Female_26_ polygamous]

The fear of losing power over women by men was frequently mentioned as an aspect that restricts men from supporting their wives’ economic opportunities i.e. VICOBA, and consequently can encourage women to engage in extramarital affairs: *“Usually men when you tell them about VICOBA, they do not want to hear about that, they do not like to be asked about money to cater for VICOBA, they think that when you get money you will be at the same level with them, they just like women to depend on them for everything. That is why women here (in this community) decide to look for an extramarital partner who will assist them to keep up with VICOBA”* [FGD_Women_01]

#### Income

Having good income was one of the main aspects reported by participants which facilitate men’s engaging in extramarital affairs. One participant pointed that it is a privilege for a man with income to have several women:

*“Us men when we get money we feel privileged to have several women […] it speaks about the status [….]and I tell you any man with high income will not be complete without having several women”*

[IDI_Male_63years_Polygamous]

#### Alcohol

Participants were in the opinion that alcohol consumption encourages extramarital behaviors of men and women through sexual networks in the local brew. It is believed that for men excessive alcohol stimulate their sexual desire and make them bold enough to approach several women at the alcohol venues. It was mentioned that this behavior could also be experienced by women.

Participants thought that exposure to extramarital relations at the local brew could be resulting from failure of men and women to be accompanied by their marital spouses when visiting the local brews. Men and women may not feel comfortable visiting the local brew with their spouses, since this could infringe their freedom to socialize. Women mostly participate in local brewing, while men are the main customers:

*F: What happens in the local brew with regards to extramarital affairs?*

*P: For men alcohol goes down to the penis and they no longer feel shy [when they have consumed excessively]. You know, when you are in the local brew you can get a woman and take her for sex, even women when they are drunk they behave the same (having extramarital affairs)*

*F: What happen to a woman in the local brew?*

*P: Women sell the local brew and we (men) buy alcohol for them and other friends*

*F: Does this apply also for married women?*

*P: Let me tell you my sister, it is rare here to find married couples going together to the local brew since this will limit their socialization, enjoyment and searching for extramarital partners […]*

[IDI_Male_30years_Monogamous]

### Marital relationship quality

#### Marital conflict

Participants’ views (mostly women) indicated that as much as extramarital affairs seems not to be a good habit, uncertainties within the family may force one to look after an extramarital partner who will provide social and psychological comfort: *“It (extramarital affairs) is not a good habit but what you do because there are so many problems in the family and sometimes you may even feel like committing suicide, so you just decide to look for someone who will provide money for food and make you comfortable and maintain your peace”*

[IDI_Female_41 years_Monogamous]

#### Sexual dissatisfaction

Male and female participants raised their concerns that relates to marital sexual dissatisfaction, which could also encourage them to search for extramarital partners. While comparing the quality of sex they receive from their extramarital partners and that which they receive from their husbands, some women complained that they are being denied ‘romancing’ including not being prepared enough for sex by their husbands: *“[…], My husband just comes and start making love as if he is entering the farm [..] but the one from outside (extramarital partner) start talking to me for a long time [..]. I* ***just*** *do sex with my husband just because he is my husband […]”* [IDI_Woman_26_Monogamous]

Men complained about being denied opportunity to practice as much sex as they would wish by their wives. This aspect was mentioned mainly by men as one of the reasons for them to search for extramarital partners:

*“You find that I need sex frequently or I have the speed more than my wife but she refuses to give me whenever I need (sex) and then I need to look for other women […]”*

[IDI_Male_30years_Monogamous]

## Discussion

The study findings suggest that extramarital affairs in Ifakara marriages are socially constructed through multiple complex contextual (social-cultural) determinants and pathways. Hence, we think that extramarital affairs should not merely be regarded as ‘risky behavior’ or be linked to economic factors alone, but it is important to appreciate the multiple social-cultural factors that contributes to the behaviour. This observation collaborates with findings from similar studies in Tanzania [4, 12, 13] and outside Tanzania [15] where multiple factors were found to influence extramarital affairs for men and women.

The qualitative findings in this study further revealed that although married men and women’ extramarital affairs could be influenced by similar factors, but the pathways seem different. In this case, we think that addressing the drivers for extramarital affairs for partners in Tanzanian marriages, requires a deeper understanding of the context and the pathways that influence the behavior, and not just instructing married partners to abstain from extramarital affairs. A better understanding of the pathways in which married partners engage in extramarital affairs can help improve the existing HIV prevention programs for men and women in the country. Various HIV prevention interventions have been in existence for long, however, HIV vulnerability remains substantially high in this group. One of the pathways that could be considered is ‘networking in VICOBA groups’. HIV programmers could use VICOBA (the microfinance groups) as platforms for mainstreaming HIV prevention strategies among married partners. We think that these are platforms where economic empowerment programs could be implemented together with HIV awareness creation, skills building on safer sex negotiation skills and couple dialogues on harmful social norms and gender inequality aspects that makes men and women vulnerable to HIV infections.

As found in another study conducted in the same context [8], the current study also supports the importance of incorporating additional social constructs; i.e. relationship quality and social beliefs, into the WHO-Social determinants of health framework (Fig. 1) in order to denote a broader meaning of extramarital affairs in marriage.

### Gender inequality dimension of extramarital affairs as linked to HIV infection

In the case of women, in this study extramarital affairs were associated with a higher HIV prevalence. Specifically, we observed a significant association between lifetime extramarital affairs and HIV status among women only. It is worth pointing out that having more HIV infections among women than men is not new in SSA including in Tanzania [29, 30]. However, the finding that an extramarital affair has a significant association with HIV status among women only, is peculiar since the common understanding is that both men and women are at the risk of acquiring HIV infection through risky sexual behaviors. The finding is also contrary to other observations that men more often engage in extramarital affairs and thus are at risk of HIV infection [12].

Although biologically an increased HIV vulnerability among women exists [31], the study observations of the significant association between extramarital affairs and HIV status among women only, may also lend support to the view that beyond biological vulnerability, social-economic constraints including gender inequality in access to economic opportunity [13] and safer sex negotiation [8] potentially subject women including married women to high risk of contracting HIV infections if they engage in extramarital affairs. The qualitative findings found in this paper also support the above elucidations, as they reflect that economically vulnerable married women in the study community may seek money from extramarital partners to support their VICOBA business to make a living. In such a context, women may have less power to bargain for condom use during the extramarital sex and this can increase their risk of contracting HIV if the partner is already infected. DeWalque in SSA including in Tanzania found that married women who engage in extra-marital sex are less likely to use condoms than single women when doing so [6]. Studies in Zambia [9] and Tanzania [8] found as well that married women are not often given rights to decide on HIV related matters including safer sex initiation such as condom use.

### Social-structural associates of extramarital affairs of men and women in Ifakara town

#### Being a member of VICOBA

The study findings revealed that being a member of a VICOBA was statistically significantly associated with the relative risk of engaging in extramarital affairs for men and women.

To the best of our knowledge, this finding is new in the respective literature and requires further investigation. But we have tried to discuss the finding in the light of information drawn from the qualitative findings and from the validation sessions conducted in the study community. What we know is that, VICOBA as an informal social protection mechanism in Tanzania is expected to improve men and women’s financial saving capacity and enhance their economic livelihood [32, 33]. How VICOBA exposes women to extramarital practices is an interesting question especially at this era when social protection mechanisms are expected to alleviate women’s poverty and HIV vulnerability [34]. The qualitative findings in this study suggest that women with economic hardship in Ifakara town are likely to have several debts in VICOBA since they are unable to raise enough money to support their membership in VICOBA groups. During the validation sessions of the qualitative data [as described in the analysis section of qualitative study in this paper], participants further clarified that, some women belonged to several VICOBA groups, which in turn brought them in the situation that they couldn’t keep up with paying all the loans. Our findings further revealed that some husbands may refuse to support their wives’ VICOBA membership fearing loss of power and competition in the households even in the context where men would in turn borrow money (received from VICOBA by their women). This may imply that, engaging in extramarital affairs seems to be an acceptable coping strategy for women who are economically dependent on men. A similar study in Tanzania found that women may engage in multiple concurrent sexual partnership to ensure social and financial security for their families, as such they nick-named a concurrent sexual partnership as ‘mafiga matatu’ (three stones used to support fire wood when cooking) [13]. This symbolic meaning of an extramarital affair is similar to that which we found in our study that ‘Kidumu’ was also a local name for an extramarital partner of women in Ifakara town and was considered as a financial back up in case one fails to meet the demands of VICOBA.

In the case of men, the qualitative findings further clarified that, men in a VICOBA usually found opportunities to establish sexual networks with non-marital spouses since it is unlikely that both spouses belong to the same VICOBA group. This finding can be supported by Comas’ observations [35] that men tend to form extramarital relation in dense networks. Although in this study we had fewer men (4% men vs 14% women) reporting belonging to VICOBA, it could be that men have sexual relations with several women in the same VICOBA group.

In the view of the above highlights, it seems that VICOBA groups could be an important platform to foster HIV prevention strategies for married men and women in the study community.

### Alcohol consumption

In this study alcohol consumption was statistically significantly associated with engaging in extramarital affairs for married men and women. Alcohol consumption for married men and women in Tanzania is not uncommon. However, excessive alcohol consumption can be detrimental to an individual’s general health and can also expose someone to sexual networking and subsequent risks when visiting alcohol brewing and selling venues. The qualitative findings showed that most of the extramarital sexual relations in Ifakara town happened in the local brew places. Visiting alcohol brewing and selling venues could be especially risky for married couples since our qualitative findings showed that it is uncommon for married couples in Ifakara to go together to the local brew venues. In absence of the spouse there is a chance that married men and women who become drunk are more likely to initiate sexual affairs. Several studies in Tanzania have shown positive association of alcohol consumption and extramarital affairs for both women and men [36], [37]. However, in the Ifakara context women in alcohol venues may be more vulnerable to risky sexual behaviors since the qualitative study suggests dual pathways to exposure, first by being customers at the local brewery, secondly by being the main sellers of local brew.

### Re-married

Our study also shows that the relative risk of exposure to extramarital affairs was significantly higher among participants who reported to have been re-married. In parallel, a study by deWalque in SSA and Mtenga in Tanzania showed that married men and women who reported to have been re-married were significantly more likely to have been HIV infected [38].

Our observation could imply that women who leave their husbands for subsequent marriages or those who have been widowed or left by husband are likely to possess self-autonomy in trying several extramarital relations to reach for better-committed partnerships. However, this may need further investigation.

### Being from southern parts

An observation that women from southern parts of Tanzania were at relative higher risk of engaging in extramarital affairs than those from other parts is consistent with other data from Tanzania. Generally, the southern parts of Tanzania retain substantial level of HIV infections. [29]. A significant association between being from southern region and extramarital affairs among women is interesting and could be partly due to migration and mobility which are among the structural aspects associated with HIV risk behaviors including having multiple sexual partners. An evidence from Tanzania indicated that patterns and conditions of mobile population in relation to their requirements of each different economic activity influenced the nature of relationships that mobile groups have, how and where local sexual networks are accessed, and the practicalities of having sex. The authors pointed that this trend has further implications for condom use [39]. Another study among the key populations in Tanzania indicated that the long-truck drivers were potentially at risk of engaging in higher risk sex (not using condoms during heterosexual anal sex) [20]. Experience and observations shows that several women from southern parts of Tanzania usually engage in various businesses with various traders from inside and outside the country. They can also have constant interactions with long-distance truck drivers (mostly buying rice and banana), this could expose them to various sexual networks. This specific finding support that, the southern part of Tanzania remains to be a target for intensive HIV prevention interventions.

### Gender (being a man)

The finding that men were significantly more likely to engage in extramarital affairs than women is not new in SSA. A study in Nigeria found also that husbands [14] practice most of the infidelity behaviors. Men’s practice of extramarital affairs could be culturally oriented and tolerated since the qualitative component revealed the existing beliefs in Ifakara town which justify men’s possession of multiple women as a prove of their manhood as well as a support of perceived religious belief that ‘a man should dominate a woman’. Furthermore, a societal expectation that men have the right to possess multiple women is possibly connected with the patriarchal system which considers women as subordinates and men superior [41, 42]. Possession of multiple women by men could be one way to prove this superiority. At the same time, men are more likely in the position to enforce condom use.

### Being at older age

The study findings further suggest that extramarital affairs significantly decreased with age increase for men and women. This potentially reflects that older age can be protective against extramarital affairs. Mbago et al. [12] observed a similar pattern in southern Tanzania. However, this could be context-specific since a study in Cameroon found that extramarital practices significantly increased with older age [40].

### Relationship quality (sexual dissatisfaction and conflict)

Issues of relationship quality were frequently cited during qualitative study where conflicts, and sexual dissatisfaction emerged as recurred themes. These aspects were reported to encourage either partner to engage in extramarital affairs. These observations could imply that relationship uncertainties in marriage could foster extramarital affairs and that a good relationship quality could reduce the occurrence of behavior. In Zambia, a good relationship quality was found to prevent married men and women from unsafe sex practices [43]. In Kenya, sexual satisfaction was found to be protective against extramarital partnerships among married women [44].

## Conclusion

The study findings suggest that extramarital affairs transcend the notion that it is just a ‘risky behavior’ and that multiple contextual aspects are likely to contribute to the behavior. The findings suggest that the link between HIV and extramarital affairs has a gender dimension in which women can be more exposed to extramarital affairs and HIV infection based on various gender dynamics including women’s economic hardship, social beliefs and masculinity norms. This view can be support by another research done in the same sample which revealed that, the level of HIV prevalence among women was significantly higher as opposed to that of men [24].

The multiple associates of extramarital affairs for men and women, differences in the pathways to engaging in the behavior, and the peculiar finding such as the positive association between VICOBA and extramarital affairs, suggest a need to understand and address the multiple drivers of extramarital affairs while considering the context-sensitive interventions. Religions through spiritual messages, national and community events such as meetings could be used as an entry point to promote women’s empowerment, economic independence, marital fidelity, relationship quality, and discourage harmful social norms of masculinity. VICOBA in Ifakara town could be tailored to provide economic and HIV risk protection as well as gender-transformative approaches to HIV prevention.

## Limitation

The study had several limitations. First, the study was cross sectional i.e. exposures and HIV status had been determined at the same time. Second, we did not have data for condom use which could have enabled the association with extramarital affairs. Third, two-thirds of the married or cohabiting respondents in the MZIMA cohort are female, which means that our study sample is not very much representative of the male/female distribution in the study area. Fourth, the variables measuring extramarital affair could have a reporting bias and the proxy for extramarital affair more than 12 months ago may lack accuracy. We assume that the proxy for life-time extramarital affairs are likely to have overestimated the prevalence of extramarital affairs since it was calculated from the average number of multiple sexual partners at age of marriage. This might have included some of the relationships, which happened before marriage. Therefore, the results should be interpreted with caution by accounting for the possible overestimation of the outcome variable. However, the triangulation of data from the mixed method approach suggests quite a good internal validity for most of our quantitative observations. For example, the qualitative data provided various life scenarios that supported the association between VICOBA and extramarital affairs for women. Likewise, most of the narratives pointed to various social construct contributing to men and women’s extramarital behaviors. Most of our findings are further supported by studies from other SSA countries. For example, in our data we observed the association between income, alcohol and extramarital affairs among men and women, which has been also observed in other contexts. However, further research on how sexual behaviors exposes women to HIV infection, and marital relationship quality is needed to gain a better understanding of the epidemic.
